# An Innovative and Efficient Diagnostic Prediction Flow for Head and Neck Cancer: A Deep Learning Approach for Multi-Modal Survival Analysis Prediction Based on Text and Multi-Center PET/CT Images

**DOI:** 10.3390/diagnostics14040448

**Published:** 2024-02-17

**Authors:** Zhaonian Wang, Chundan Zheng, Xu Han, Wufan Chen, Lijun Lu

**Affiliations:** 1 School of Biomedical Engineering, Southern Medical University, 1023 Shatai Road, Guangzhou 510515, China; hxqwzn@gmail.com (Z.W.); zhengchund@outlook.com (C.Z.); hanxv8826@gmail.com (X.H.); 2 Guangdong Provincial Key Laboratory of Medical Image Processing, Southern Medical University, 1023 Shatai Road, Guangzhou 510515, China; 3 Guangdong Province Engineering Laboratory for Medical Imaging and Diagnostic Technology, Southern Medical University, 1023 Shatai Road, Guangzhou 510515, China; 4 Pazhou Lab, Guangzhou 510330, China

**Keywords:** computer-aided diagnosis, survival analysis, PET/CT, multi-modal fusion, head and neck cancer

## Abstract

**Objective:** To comprehensively capture intra-tumor heterogeneity in head and neck cancer (HNC) and maximize the use of valid information collected in the clinical field, we propose a novel multi-modal image–text fusion strategy aimed at improving prognosis. **Method:** We have developed a tailored diagnostic algorithm for HNC, leveraging a deep learning-based model that integrates both image and clinical text information. For the image fusion part, we used the cross-attention mechanism to fuse the image information between PET and CT, and for the fusion of text and image, we used the Q-former architecture to fuse the text and image information. We also improved the traditional prognostic model by introducing time as a variable in the construction of the model, and finally obtained the corresponding prognostic results. **Result:** We assessed the efficacy of our methodology through the compilation of a multicenter dataset, achieving commendable outcomes in multicenter validations. Notably, our results for metastasis-free survival (MFS), recurrence-free survival (RFS), overall survival (OS), and progression-free survival (PFS) were as follows: 0.796, 0.626, 0.641, and 0.691. Our results demonstrate a notable superiority over the utilization of CT and PET independently, and exceed the result derived without the clinical textual information. **Conclusions:** Our model not only validates the effectiveness of multi-modal fusion in aiding diagnosis, but also provides insights for optimizing survival analysis. The study underscores the potential of our approach in enhancing prognosis and contributing to the advancement of personalized medicine in HNC.

## 1. Introduction

Investigating intra-tumor heterogeneity in head and neck cancer (HNC) has substantial implications for advancing outcome prognostication and individualized treatment planning [1,2]. Surgery, chemotherapy and radiotherapy are the main treatments for HNC, and great progress in these fields has been made in recent years, which has improved the long-term survival rates of early-stage patients to approximately 70–90%. For diagnostics in the HNC, Positron Emission Tomography (PET) and Computed Tomography (CT) are two crucial and commonly used medical imaging modalities in HNC clinical application scenes [3]. PET images primarily focus on the metabolic characteristics of tumor regions, while CT images provide valuable anatomical structural information [4]. The combination of these two modalities yields a more comprehensive and clinically relevant performance, leading to enhanced performance and outcomes for precision medicine in clinical applications.

Therefore, how to efficiently integrate the information between PET and CT, making the best possible use of the information characteristics of different modal images, and making up for the dimensional differences between the images is a very essential part of the promotion of images in the field of precision medicine diagnosis, and needs to be solved urgently. However, there is still debate regarding how to efficiently utilize the information from both modalities. A common approach is to use multi-modal deep learning networks that can simultaneously process PET and CT images and extract useful features [5]. These networks can include components such as Convolutional Neural Networks (CNNs) [6,7] to effectively capture different features from PET and CT images. Another approach is to employ feature fusion techniques, such as feature-level fusion or decision-level fusion. Feature-level fusion combines the features of PET and CT images to generate a more comprehensive feature representation. Decision-level fusion combines the prediction results of PET and CT images to obtain more accurate outcomes [8]. In addition, other methods like attention mechanisms have been explored to improve the fusion of PET and CT modalities [9,10,11] to gain the final result. Therefore, an elegant fusion method of PET and CT that can be tailored to specific tasks is required, and is as yet lacking.

In the realm of clinical auxiliary diagnostic tasks, clinical textual information holds equal significance. Key clinical textual details, including factors like tumor node metastasis (TNM) classification and gender, play a pivotal role in the diagnosis and prognosis of HNC. The prognosis of HNC is predominantly dependent on the TNM staging system, tumor location, and the chosen treatment approach [12,13]. However, the TNM stage relies solely on anatomical information, posing limitations in capturing the biological heterogeneity of HNC. Consequently, there is an urgent need to identify more informative prognostic factors and establish robust risk stratification tools for precise and personalized treatment strategies. Moreover, understanding how to integrate case information, presented in a textual form corresponding to TNM, into our prognostic and diagnostic models is crucial. Previous approaches have often neglected the true numerical significance of this information by encoding it without considering its clinical meaning. Assigning numerical values to different textual features in a simplistic manner is not a reasonable approach, as there is no numerical significance between them. Additionally, directly concatenating features from textual and image information is not feasible due to dimensional differences between the two data types. Therefore, it is imperative to explore alternative methods that capture the true meaning of clinical textual information and address dimensional disparities between textual and image data, aiming to achieve optimal results in integrating these two types of information.

Beyond the optimization of information fusion, such as PET, CT, and text information, it is imperative to systematically identify and employ pertinent metrics and models. This strategic approach is essential for ensuring the attainment of results that exhibit high relevance to the field of precision medicine. Against this background, the survival models based on deep learning and PET or CT can adaptively learn high-level features from images with high predictive performance and realize end-to-end functions, which have gradually become the mainstream of deep survival models [14,15,16].

In the realm of therapy and diagnostics in HNC, medical researchers often utilize survival models to assess the significance of prognostic variables in outcomes such as death or cancer recurrence. This information is then used to inform patients about their treatment choices, thereby contributing to the development of precision medicine in the context of HNC. For the previous work, Yin et al. [17] developed a concise and efficient survival model based on deep learning, which has achieved more robust and superior prediction accuracy when applied to various cancers compared with the state-of-the-art survival analysis model. Lv et al. used the radiomics features from PET and CT to gain a multi-modal result [18]. Although deep learning and other machine learning methods have achieved acceptable performance in HNC survival prediction, a major limitation still exists, as seen in related work based on the Cox proportional hazards model [19,20]. The optimization of conventional survival analysis Cox models has emerged as a pivotal research direction in relation to gaining more precise results.

In summary, within our innovative HNC deep learning model, we have introduced a novel approach by bifurcating this section into “muti-modal fusion” and “tailored survival analysis “components. The first involves PET, CT and textual information; here, we seamlessly and efficiently integrate PET, CT, and textual information. The second component entails improvements in the survival analysis prediction model. We transcend traditional Cox prediction models by incorporating temporal variables into the relevant predictive models, ultimately yielding results of heightened precision. To address the above issue, there is an imminent need to develop a deep learning model that can efficiently utilize PET, CT, and related tumor clinical text information, and can be used to perform survival analysis on patients with high performance. The implementation of the multi-modal approach, the performance of SSL, the improvement of the survival analysis scheme and the validity of our proposed approach will be presented in detail, and these will demonstrate the validity of our proposed approach through detailed and corresponding experiments and results.

The code can be obtained from https://github.com/YahooKID/HNCmodel URL (accessed on 5 December 2023).

Specifically, our work offers the following three innovations:

1. A multi-modal information utilization scheme for transformer-based image-text fusion has been developed, which can utilize not only image information but also the corresponding text case information;

2. To achieve modality-specific modeling results for PET and CT images, a self-supervised learning cross-attention approach is implemented for the fusion of distinct medical image modalities;

3. We improved the prognostic scheme of traditional survival analysis by not limiting ourselves to the Cox regression model and its customary assumptions, but also developing a deep learning-based survival analysis model with stronger fitting capabilities.

**Figure 1:**
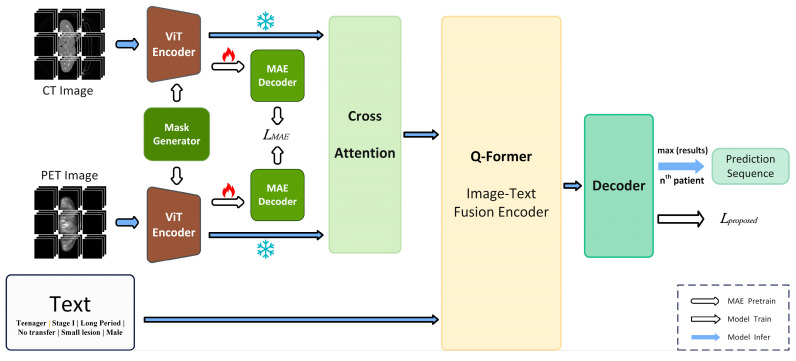
The work flow of the whole model.

The entire process of the experiment is demonstrated in Figure 1. The Cross attention architecture integrated the features of PET and CT, while the Q-former architecture integrated the features between text and image. The subsequent information was predicted using the decoder architecture to obtain corresponding results.

## 2. Dataset

We utilized multi-center head and neck cancer (HNC) data from The Cancer Imaging Archive (TCIA); the link to the datasets are https://wiki.cancerimagingarchive.net/display/Public/Head-Neck-Radiomics-HN1 (accessed on 10 October 2023), https://wiki.cancerimagingarchive.net/display/Public/QIN-HEADNECK (accessed on 10 October 2023) and https://portal.gdc.cancer.gov/projects/TCGA-HNSC (accessed on 10 October 2023). The inclusion criteria for the data are as follows:

1. Histologically confirmed HNC;

2. No distant metastasis at the time of initial diagnosis;

3. Availability of complete follow-up information;

4. Availability of pre-treatment PET/CT images.

The exclusion criteria were as follows:

1. Receipt of anti-cancer treatment before PET/CT imaging;

2. History of HNC or other cancers.

HNC data were collected from seven different centers: The Head-Neck-PET-CT dataset included 296 patients from four centers in Canada; 100 patients were from Centre hospitalier universitaire de Sherbrooke (CHUS), 65 were from Centre hospitalier de l’Université de Montréal (CHUM), 90 were from Hôpital général juif (HGJ) de Montréal, and 41 were from Hôpital Maisonneuve-Rosemont (HMR) de Montréal [18]. The Head-Neck Radiomics-HN1 dataset included 74 patients from MAASTRO Clinic in the Netherlands [21]. The TCGA-HNSC dataset included 29 patients from the University of Pittsburgh, and was denoted as TCGA [22]. The QIN-HEADNECK dataset, including 243 patients from University of Iowa, is denoted as QIN [23]. In this study, 482 patients from four centers were used for model construction and validation, in which 80% of patients were randomly selected from each center and were grouped for training, while the remaining 25% of patients were used for testing the model’s effectiveness. This means that 482 patients (QIN—243, CHUS—100, CHUM—65, HGJ—74) were selected for the training set, while 70 patients (CHUS—70) were selected for the internal validation set. Moreover, 160 patients from four independent centers (HGJ—16, HMR—41, TCGA—29, MAASTRO—74) were selected for the external testing set.

1. We collected and organized patient characteristics, along with corresponding follow-up information, from structured text or DICOM Structured Reporting (SR) files. Clinical information included age, gender, stage I, stage N, and TNM stage.

2. The primary experimental endpoint of this study is overall survival (OS) [24], defined as the time from diagnosis to the first occurrence of confirmed recurrence, metastasis, death, or a combination of these events (whichever occurs first). The additional three endpoints include recurrence-free survival (RFS) [25], metastasis-free survival (MFS) [26], and progression-free survival (PFS).

For patients with fully delineated DICOM segmentation results (SEG) or radiation therapy structure data sets (RTSTRUCT), the total tumor volume of the primary tumor and lymph nodes (GTV and GTVnd) is extracted as the segmentation delineation result for CT. This result is then mapped onto the corresponding PET image to generate the corresponding PET delineation result. Otherwise, the PET segmentation delineation result is semi-automatically delineated by a radiologist expert (HZ) with four years of experience in head and neck nuclear medicine. The delineation module used is based on the “level tracing” algorithm in 3D Slicer 4.10.2 (https://slicer.org/, accessed on 3 November 2019). The corresponding delineation target area on CT is generated based on the corresponding registered image.

PET/CT imaging exhibits variations in terms of manufacturers, scanners, acquisition protocols, and voxel sizes. For the PET images, the manufacturers GE, Philips, Siemens, and CPS scanned 337, 144, 182, and 143 patients, respectively. The scanner types included GE Discovery (RX, ST, STE, and LS), Philips (Amsterdam, Netherlands) GeminiGXL 16, Siemens (Berlin, Germany) (Biograph TruePoint 1093, Biograph 40 TruePoint, Biograph 64 mCT, and Biograph SOMATOM Sensation-16), and CPS (1023, 1024 Biograph HiRes Model Biograph Hire 1080).

Regarding CT images, GE, Philips, and Siemens scanners from three different brands scanned 206, 276, and 324 patients, respectively. The scanner types included GE Discovery (LS, RX, ST, STE, and LightSpeed RT16), Philips (GeminiGXL 16, AcQSimCT, Brilliance 64, Mx8000 IDT 16, and PQ5000), and Siemens (Sensation 16, Emotion Duo, Biograph SOMATOM Sensation-16, Biograph 64, and Biograph 40).

The plane resolution of the PET images used in this study ranged from 1.95 to 5.50 mm, while the plane resolution of the CT images ranged from 0.48 to 1.95 mm. The slice thicknesses for PET and CT varied between 2.0 and 5.0 mm and 1.5 and 5.0 mm, respectively. Specific results and their corresponding clinical analyses are presented in Table 1.

We performed numerical normalization and bandpass filtering as preprocessing operations on the data to remove abnormal noise points and spurious signals corresponding to the images. Specifically, we applied a bandpass filter with critical values of 1‰ and 10% to the images, resulting in the final dataset used for network training and inference.

## 3. Methods

To harness a larger dataset, an SSL approach combined with textual information was employed. SSL effectively addresses the extraction of data, enabling the acquisition of more extensive datasets. However, a pivotal question arises regarding the utilization of such information. We adopted the QFormer, integrating image features and textual information. Nonetheless, due to the limited availability of labeled data, concurrently training the image encoder and QFormer induced a susceptibility to overfitting.

To mitigate this challenge, cross-attention was introduced to selectively filter and transform the abundant image features extracted by the image encoder. This intermediary process effectively allows for simultaneous focus on salient information (leveraging the attention mechanism) and the further transformation of feature maps (leveraging the network’s intrinsic capabilities). This approach facilitates more effective training. However, conventional loss functions impose constraints on the network’s output space. Therefore, the sigmoid function, which is classic and was deemed suitable for the task at hand, was selected. This choice further refined our model, laying a foundation for the successful completion of the task.

We will outline the algorithmic details of each part in the following sections.

### 3.1. Self-Supervised Learning (SSL) for Feature Extractor

First, to extract precise and refined features of the PET and CT, the SSL [27,28] scheme was used for pre-training to develop a feature extractor for PET and CT images. The mask ratio was 75% and the backbone of the model was a Vision transformer. We employed the MAE (Masked Autoencoders) [29] approach to independently pretrain PET and CT images in parallel, aiming to restore the image information between the two modalities. In clinical scenarios, the issue of modality mismatch may manifest in the context of practical applications involving PET and CT. For instance, not all patients undergo imaging with both modalities; some patients may exclusively undergo CT imaging. Adhering strictly to a paired-training paradigm would result in the exclusion of patients with only CT data. To maximize the utility of available data, we here employed MAE for pretraining. The adoption of MAE as a pretraining task is seen within the realm of medical imaging, where images generated through specific imaging protocols consistently manifest intricate anatomical structures housing clinically relevant information dispersed across the entire image. Notably, the discernment of high-level structural information, encompassing anatomical structures and their relative spatial orientations, holds paramount significance for the identification of normal anatomical configurations and the delineation of various disorders.

Given the exigencies of medical imaging tasks, there arises a dual requirement for the model to concurrently attend to both high-level global features and fine-grained details dispersed throughout the images. Recognition tasks in medical imaging demand the extraction of complementary discriminative features that encapsulate both the overarching anatomical context and the subtle, localized variations in texture crucial for tasks such as disease identification, organ delineation, and lesion isolation.

In this context, the utilization of MAE as a pretraining task aligns with the inherent capacity of autoencoders to capture hierarchical representations. By incorporating a masking mechanism within the MAE architecture, the model is poised to effectively discern and learn salient features within medical images. This strategic integration of masking in the MAE training process enhances the model’s ability to encapsulate critical features, laying the foundation for subsequent tasks such as disease recognition and organ segmentation. The resultant pretraining with MAE facilitates the generation of feature representations that exhibit a nuanced amalgamation of high-level structural coherence and a heightened sensitivity to localized intricacies, thereby rendering the model adept for the nuanced demands of medical image analysis tasks.

We constructed a dual-stream network to extract PET/CT image features separately for respective learning processes, and obtained corresponding feature extractors for PET and CT. These extractors were subsequently utilized for modal fusion, model fine-tuning, and training. This involved the fusion of multi-scale images and multi-modal features for comprehensive integration. After the MAE pre-training, we obtained two corresponding feature extractors for the subsequent correlation analysis.

### 3.2. Multi-Modal Information Fusion for Survival Analysis Prediction

The two feature extractors dedicated to PET and CT, developed through the MAE pretraining, generated respective image features. Subsequently, a multi-modal fusion approach was applied to integrate these features, leading to corresponding predictive outcomes. It is crucial to note that the processes of MAE pretraining and modal fusion are independent of each other and do not interfere. MAE pretraining focuses on acquiring more detailed image features, ensuring that the subsequent modal fusion process remains undisturbed and facilitates the extraction of comprehensive information from both PET and CT modalities. It is important to note that not all scenarios necessitate the use of paired PET and CT images. Our approach maximizes the utilization of collected data. In this experiment, feature extractors trained through SSL pretraining did not require PET and CT pairs. SSL pretraining can be conducted individually for PET and CT without the need for specific manually annotated labels. This flexibility allowed us to obtain pretraining weights suitable for both PET and CT, thereby accelerating the convergence of subsequent networks. By adopting this approach, we were able to mitigate the issue of modality mismatch in the dataset during network training.

In the second step, we used the pre-trained features from the previous step and used a cross-attention-based [30] interactive attention approach for PET/CT feature fusion. Specifically, we used PET as the V (value) of the attention mechanism, and CT as the corresponding Q (query) and K (key). The reason is that PET contains a variety of information, including not only the metabolic information of the tumor but also the anatomical structure information, while CT is relatively monotonous and only focuses on the anatomical structural information. After the fusion of the two through the cross-attention mechanism, feature fusion was performed, and eventually, features with both PET and CT information were obtained.

**Figure 2:**
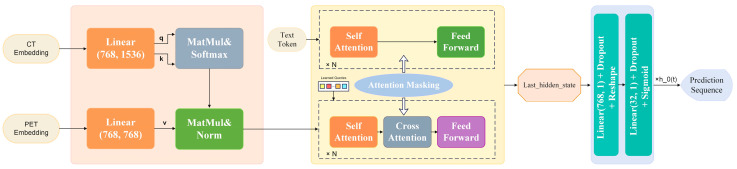
The attention-based fusion mechanism designed in our proposed model.

After that, we used Q-Former [31] for multi-modal feature fusion. Q-Former is a transformer-based contrastive learning architecture that references Blip2. It acts as a bridge between image and text information, using learnable query vectors to extract and represent visual features. Specifically, it has two information streams, namely, the image information stream and the text information stream. For the image information stream, we used feature extractors after the pre-training had been carried out in the first frozen step to obtain the deep features fused with PET/CT. For the text stream, we used TNM, age, and gender as the clinical text information in this paper. The cross-attention of the details that we used in our work is shown in Figure 2.

As shown in Figure 2, CT and PET are individually incorporated into the cross-attention mechanism, where CT passes through a linear layer to serve as the query and key, while PET serves as the value. The fusion of these two modalities through the cross-attention mechanism is then integrated into the Q-former architecture. The Q-Former is a transformer-based architecture acting as a bridge between the image and text information, using learnable query vectors to extract and represent visual features. Finally, the multimodal features were processed through a Multilayer Perceptron (MLP) to obtain the ultimate prediction results.

### 3.3. Modified Survival Analysis Model-Based Deep Learning

Having obtained an accurate feature extractor, it is also particularly crucial to use an accurate predictive model. In therapy and diagnostics in oncology, medical researchers often use survival models to assess the significance of prognostic variables in outcomes such as death or cancer recurrence, and subsequently inform patients of their treatment choices in order to facilitate the development of precision medicine. The standard survival model is the Cox proportional hazards model (CPH).

The Cox Proportional Hazards (CPH) Model, also known as Cox Regression, is a statistical technique used for survival analysis in the field of biostatistics and epidemiology. Developed by David R. Cox, this model assesses the relationship between the survival time of subjects and one or more predictor variables. Unlike parametric survival models, the Cox model does not assume a specific form for the hazard function, making it semi-parametric. It estimates the hazard ratio, which represents the proportional change in the hazard or risk of an event associated with a one-unit change in a predictor variable, while allowing for the inclusion of censored data. The model’s assumption is that the hazard functions for different levels of the predictor variables are proportional over time, providing valuable insights into the impact of various factors on the time until an event of interest occurs, such as death or relapse. This assumption is referred to as the linear proportional hazards condition. However, the linear proportional hazards condition meets a lot of issue in this area of application, so we here tried to use deep learning-based methods to enhance the survival analysis modeling fitting ability, and finally got more accurate patient prediction results that were more relevant to the needs of precision medicine. Here, we corrected the loss associated with the survival problem in training.

Specifically, the formula for survival analysis is based on:

(1) $$\lambda (\beta ;t,x)=\lambda_{0}(t)\cdot {\hat{g}}_{\beta }(x,t)$$

We defined $\lambda_{0}(t)$ manually to make sure that we got the optimal result; β is the parameter of the function ${\hat{g}}_{\beta }$ and ${\hat{g}}_{\beta }(x,t)$ is the learnable result in our model. In our application, we used the Sigmoid to fit the output of the result and endow the model with a powerful nonlinear fitting capability.

The partial likelihood was parameterized by β and defined as:

(2) $$L_{c}(\beta )=\prod_{i:E_{i}=1}\frac{\lambda_{0}(t){\hat{g}}_{\beta }\left(x_{i},t\right)}{\sum_{j\in ℜ\left(T_{i}\right)}\lambda_{0}(t){\hat{g}}_{\beta }\left(x_{j},t\right)}=\prod_{i:E_{i}=1}\frac{Sigmoid\left(N_{\beta }(x_{i},t)\right)}{\sum_{j\in ℜ\left(T_{i}\right)}Sigmoid\left(N_{\beta }(x_{j},t)\right)}$$

(3) $$\ln(L_{c}(\beta ))=\sum_{i:E_{i}=1}\ln(\frac{Sigmoid\left(N_{\beta }(x_{i},t)\right)}{\sum_{j\in ℜ\left(T_{i}\right)}Sigmoid\left(N_{\beta }(x_{j},t)\right)})$$

(4) y=F(image, features)

(5) $$l(\theta ):=-\frac{1}{N_{E=1}}\sum_{i:E_{i}=1}\left(\ln(Sigmoid\left(N_{\theta }(y_{i},t\right))-\ln\sum_{j\in ℜ\left(T_{i}\right)}Sigmoid\left(N_{\theta }(y_{j},t)\right)\right)$$

l(θ) is the loss function in our survival model, y is the tensor which we input into the model, and image, features refers to the features of the image and the embedding of the text information in the clinical application. This form of correction allows for the introduction of time variables into the calculations in the model. Parameter details have been added to the Appendix A.

### 3.4. Training Details

Regarding training details, the hardware configuration comprised an NVIDIA GeForce 4090 with 24 GB of memory. The software environment employed Ubuntu 20.04 as the operating system, and PyTorch 2.1.0 as the deep learning framework. During the pretraining phase, input images of size 224 × 224 were employed, with a batch size of 128 and training conducted over 1000 epochs. A mask ratio of 0.75 was utilized for Masked Autoencoder (MAE) pretraining, employing an AdamW optimizer with betas set to (0.9, 0.95) and a weight decay of 1 × 10^−2^. Subsequently, for the downstream tasks, training parameters included input images of the same size, a batch size of 256, and training for 300 epochs. A warm-up period of 10 epochs was implemented, with a learning rate of 1 × 10^−4^ and AdamW optimizer settings with betas (0.9, 0.999) and a weight decay of 5 × 10^−2^. The bootstrap was 1000 when we calculated the confidence interval.

These configurations and parameters are integral components of our model training pipeline, contributing to the robustness and efficacy of both the pretraining and subsequent task-specific training phases, as detailed in this academic discourse.

## 4. Results

The results of each part are shown in the following sections, and the results illustrate the effectiveness of the innovative schemes presented in our work.

### 4.1. The Result for Pretraining and the Modality of the Model Input

We carefully selected evaluation indicators (MFS, RFS, PFS, OS) related to survival analysis to comprehensively demonstrate the effectiveness of our model, including metastasis, recurrence, and overall survival. The specific details of these indicators are as follows, and are shown in Table 2:

OS [25]—A term used in oncology and clinical trials to refer to the length of time from either the date of diagnosis or the start of treatment for a disease, such as cancer, for which patients diagnosed with the disease are still alive;

DFS [26]—The time from random assignment to cancer recurrence or death from any cause;

RFS [26]—The time from treatment of disease to any event, irrespective of cause, except for any second primary cancers;

MFS [32]—MFS was defined as randomization related to the first evidence of distant metastatic disease or death from any cause, or was censored at the date of last follow-up;

Based on Table 2, the following results can be inferred regarding the integration of PET and CT modalities for survival analysis prediction. The details of the backbone and training pipeline have been added to the Appendix A.

The PET + CT modality with the cross-attention mechanism achieved the highest C-index (MFS, OS, RFS, and PFS), indicating superior accuracy in predicting the ordering of survival times. Furthermore, this modality exhibited lower prediction errors (MAE-Pretrain, MFS, RFS, PFS, and OS) compared to using PET or CT alone.

Specifically, the integration of PET and CT modalities with cross-attention (Attention: Yes) led to the highest C-index of 0.796 (95% CI: 0.737–0.851), indicating a better predictive performance compared to other modalities. This modality combination also led to lower MAE (0.626, 95% CI: 0.574–0.670), MFS (0.641, 95% CI: 0.582–0.690), RFS (0.691, 95% CI: 0.645–0.744), PFS (0.691, 95% CI: 0.645–0.744), and OS (0.691, 95% CI: 0.645–0.744) values, suggesting improved accuracy in survival prediction.

The utilization of cross-attention for multi-modal information fusion had a moderate impact on the results, while the combination of PET and CT modalities consistently outperformed single-modal approaches. In contrast, using PET or CT alone with self-attention (Attention: Yes) resulted in lower C-index values of 0.730 (PET) and 0.742 (CT). Although these modalities showed moderate predictive performance, the use of an integration of both modalities still outperformed them. The MAE, MFS, RFS, PFS, and OS values were also higher for PET and CT alone compared to the PET + CT modality with cross-attention.

Additionally, when cross-attention was not employed (Attention: No), the PET + CT modality exhibited a slightly lower C-index of 0.777 (95% CI: 0.715–0.839) compared to the PET + CT modality with cross-attention. This suggests that the incorporation of cross-attention improves the predictive accuracy of the model.

Overall, the findings indicate that integrating PET and CT modalities through cross-attention leads to enhanced survival analysis predictions. The combination of these modalities enables the model to capture complementary information from both imaging techniques, resulting in improved performance compared to using either modality alone. The use of attention mechanisms, particularly cross-attention, further enhances the predictive accuracy of the model. These insights highlight the importance of multi-modal fusion and attention mechanisms in optimizing deep learning models for survival analysis in medical research. We here see the benefits of integrating PET and CT modalities for use in survival analysis prediction, as this improves the accuracy and robustness of the models. The results support the notion that multi-modal information fusion enhances the understanding of complex medical data and aids in more accurate prognostic assessments.

### 4.2. Effectiveness of Multi-Modal Feature Fusion Strategies

Based on the presented table, we can draw the following conclusions: The integration of multimodal information has a significant impact on prognosis prediction in survival analysis. Among the listed models and structural configurations, the Q-Former model incorporates multi-modal textual information as its input. For prognosis prediction tasks, the Q-Former model combines multiple modalities, including CT and PET, and employs attention mechanisms. By comparing the results of different configurations, it is evident that the Q-Former model utilizing multi-modal information, via the fusion of CT and PET modalities, achieves the highest C-index value (0.796) and demonstrates lower prediction errors (MAE-Pretrain: 0.626). This indicates that the integration of multi-modal information improves the predictive performance in survival analysis. Table 3 explains the different methods of features fusion used in attention schemes, and presents the results of different model and structural configurations derived during external testing, using the C-index as the performance metric.

Furthermore, the utilization of attention mechanisms enhances the model’s accuracy to some extent, particularly when employing cross-attention mechanisms in the Q-Former model. However, the inclusion of multi-modal information remains crucial for achieving superior predictive accuracy. Table 4 shows the results with different losses.

In summary, the integration of multi-modal information, specifically the fusion of CT and PET modalities, in conjunction with attention mechanisms, plays a pivotal role in enhancing the prognostic prediction performance of the Q-Former model. These findings underscore the importance of leveraging multi-modal information and attention mechanisms in academic research to enhance survival analysis predictions. In summary, Table 4 illustrates how different combinations of loss configurations impact the model’s performance across various survival analysis metrics during external testing. The inclusion or exclusion of Cox loss and the use of the proposed loss function and focal loss contribute to variations in the C-index values, providing insights into the utility of different loss configurations in the experimental results.

### 4.3. The Effectiveness of Our Proposed Losses in Survival Analysis

Analyzing the table, we can observe the impact of the proposed loss configuration on the results. The proposed loss, when used in combination with the testing set, consistently improves the performance metrics compared to other loss configurations.

When comparing the Cox loss configuration to the proposed loss configuration, we find that the proposed loss consistently achieves higher C-index values across all survival endpoints (MFS, RFS, PFS, and OS). The C-index ranges from 0.781 to 0.796 for the proposed loss, while the Cox loss ranges from 0.731 to 0.851. These results indicate that the proposed loss leads to more accurate predictions of survival outcome.

Moreover, when considering the prediction errors (MAE-Pretrain), the proposed loss consistently outperforms the Cox loss. The values of MAE-Pretrain range from 0.619 to 0.628 for the proposed loss, while they range from 0.573 to 0.674 for the Cox loss. These results suggest that the proposed loss configuration reduces the prediction errors and improves the overall accuracy of the survival analysis predictions.

Overall, the findings highlight the effectiveness of using the proposed loss configuration in improving the predictive performance of the model. The proposed loss consistently achieves higher C-index values and lower prediction errors compared to the Cox loss. These results indicate that the proposed loss has a positive impact on the accuracy and reliability of survival analysis predictions.

### 4.4. The Kaplan–Meier (K-M) Curves in Our Experiment Result

In our study, Kaplan–Meier (K-M) curves [33] were plotted to analyze the survival outcomes for the MFS, OS, PFS, and RFS endpoints. The dataset was divided into high-risk and low-risk groups based on the predicted risk values, using the predicted values as the cutoff.

Separate survival analyses were performed for each endpoint, calculating the K-M estimates of the survival function for both groups. The resulting K-M curves were plotted with time on the x-axis and survival probability on the y-axis, visually illustrating the differences in survival probabilities between the high-risk and low-risk groups. Statistical tests were conducted to assess the significance of the observed survival differences.

**Figure 3:**
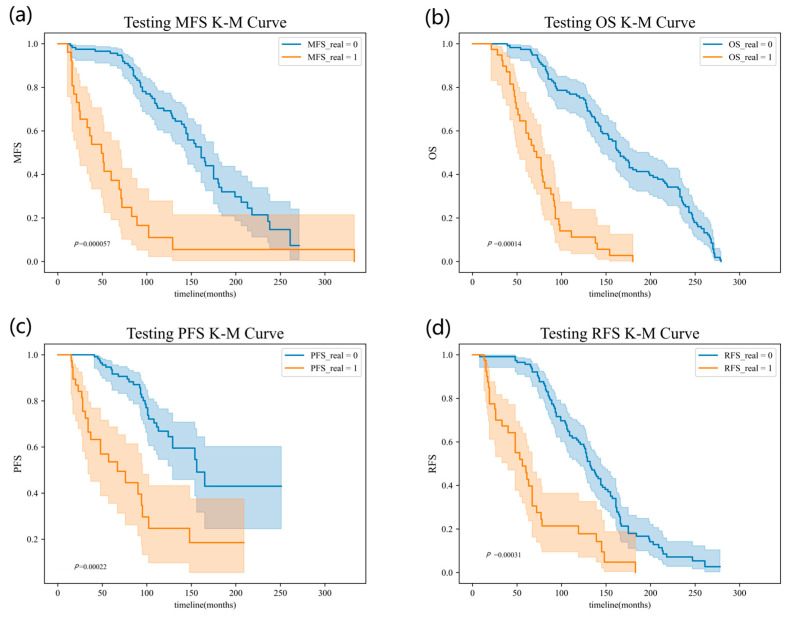
The K-M curves in our experiment setting; images (**a**–**d**) represent MFS, OS, PFS and RFS, respectively.

The K-M curves show that the model results we obtained from the whole training flow allowed for the separation of patients between high and low risks. The effectiveness of applying our model in survival analyses is again verified. The K-M curves in our experiments are shown in Figure 3.

**Figure 4:**
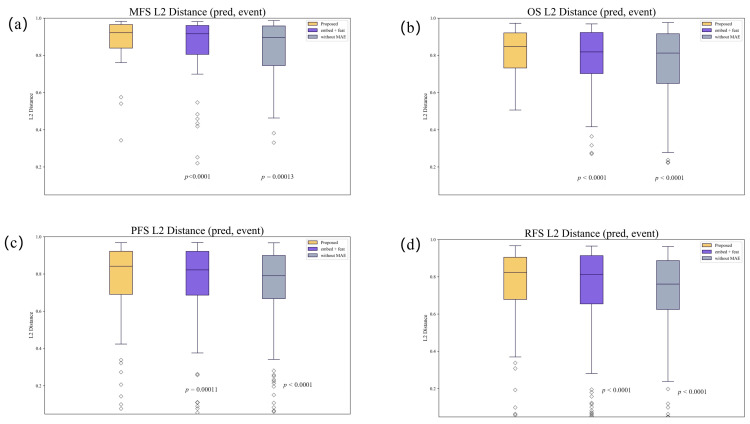
The L2 distance between the outcome predicted by the model at the moment of the follow-up event and whether the event occurred; (**a**–**d**) represent the MFS, OS, PFS and RFS, respectively.

Figure 4 demonstrates the gap between the predicted results and the real results. This distance is very narrow, proving the validity of using our model in analyzing the relationship between high and low risk and prognosis.

## 5. Discussion

This study proposes a multi-modal image–text fusion strategy to comprehensively represent intra-tumor heterogeneity in head and neck cases, effectively utilize image and text information, and facilitate the development of precision medicine. A deep learning-based model is developed, incorporating both image and clinical text information, to maximize the utilization of information. Our proposed survival analysis losses are employed to optimize model losses and training strategies, resulting in more precise survival analysis. The researchers have been exploring effective approaches to fuse the information from both modalities and align image and text information in feature dimensions. Ultimately, a multi-modal deep learning network can be obtained, which integrates information from PET and CT modalities with high accuracy, enabling the prediction and diagnosis of patient survival.

For clinical information, textual information, like TNM, is a widely validated clinical standard closely associated with tumor prognosis and diagnosis. However, there exist dimensional differences between clinical textual information and medical imaging data. Simple concatenation methods fail to achieve optimal results in network training due to these dimensional disparities. Therefore, an efficient approach is needed to effectively integrate the dimensional differences between these two types of information, aligning the features of image data and textual information in the feature space. This will enable the development of a deep learning network that can efficiently utilize both textual information and image data. Therefore, we need to explore the tumor characteristics between PET and CT images and develop algorithms tailored to their specific imaging features. These algorithms will efficiently integrate the information from both PET and CT modalities to create a fused multi-modal deep learning network. Ultimately, this network will be used to predict and diagnose the survival outcomes of patients.

Specifically, in the fusion of information of different kinds of medical images, the integration of PET and CT modalities for survival analysis prediction holds significant clinical relevance. PET provides functional information about metabolic activity, while CT offers anatomical details. By combining these modalities, clinicians can obtain a more comprehensive understanding of the patient’s condition, leading to improved prognostic predictions and treatment planning. Our previous work [34,35] also fully explored the PET/CT fusion modality and the diagnosis and prognosis of head and neck cancer in the radiomics dimension, respectively.

Integrating PET and CT modalities for survival analysis prediction may pose challenges related to data availability and integration. PET and CT scans might be acquired at different time points or by different imaging devices, leading to potential inconsistencies. Addressing these challenges requires standardized data acquisition protocols, data preprocessing techniques, and robust fusion algorithms to ensure accurate and reliable predictions.

The promising results obtained from the Q-Former model highlight the potential for further advancements in multi-modal fusion and attention mechanisms for survival analysis prediction. For integrating the clinical textual and image information, although the Q-Former model with multi-modal fusion and attention mechanisms demonstrates superior performance, it is essential to consider the interpretability of the model’s predictions. Deep learning models often act as black boxes, making it challenging to understand the underlying factors contributing to the predictions. The presented findings showcase the effectiveness of using the Q-Former model with multi-modal fusion and attention mechanisms for survival analysis prediction. It is crucial to evaluate the generalization of these results across different patient populations, healthcare settings, and diseases. Replicating the study with diverse datasets and conducting external validation can provide further evidence of the model’s robustness and applicability.

## 6. Limitation and Future Recommendations

In real-world clinical applications, the fusion of text and images should be more aligned with the clinical context [36,37]. For instance, we may need to process actual case data rather than neatly encoded clinical information. Additionally, the interpretability of deep learning is limited, and we might require corresponding interpretable approaches to better facilitate insights into clinical medical research.

Future research should explore the incorporation of additional modalities, such as genomics or clinical variables, to enhance predictive performance. Additionally, investigating novel attention mechanisms or exploring other deep learning architectures could contribute to further improvements in prognostic predictions.

## 7. Conclusions

In conclusion, our model not only affirms the efficacy of multi-modal fusion for diagnostic support, but also offers valuable insights for refining survival analysis methodologies. This research highlights the promising potential of our approach in improving prognostic assessments, thereby making significant contributions to the progression of personalized medicine within the realm of HNC.

## Figures and Tables

**Table 1:** The datasets used in our experiment for training, validation and testing.

	All	Training	Validation	Testing
No.	642	482	70	160
Age, median (range)	60 (18, 90)	60 (20, 90)	57 (39, 79)	63 (18, 88)
Sex, no. (%)				
Male	507 (79%)	379 (79%)	58 (83%)	128 (80%)
Female	135 (21%)	103 (21%)	12 (17%)	32 (20%)
TNM Stage, no. (%)				
I	26 (4%)	15 (3%)	1 (1%)	11 (7%)
II	55 (9%)	39 (8%)	6 (9%)	16 (10%)
III	121 (19%)	86 (18%)	18 (26%)	35 (22%)
IV	440 (68%)	342 (71%)	45 (64%)	98 (61%)
RFS_time, median (range)	1145 (5, 4000)	1165 (5, 4000)	164 (1100, 3402)	1102 (45, 3200)
RFS_status				
0	453 (70%)	339 (70%)	40 (57%)	114 (71%)
1	189 (30%)	143 (30%)	30 (43%)	46 (29%)
MFS_time, median (range)	1238 (6, 3351)	1265 (6, 4000)	1417 (6, 3402)	1162 (102, 3254)
MFS_status				
0	496 (77%)	376 (78%)	48 (69%)	120 (75%)
1	146 (23%)	106 (22%)	22 (31%)	40 (25%)
OS_time, median (range)	1349 (30, 4000)	1303 (30, 4000)	1417 (260, 3402)	1591 (103, 3351)
OS_status				
0	451 (70%)	355 (74%)	45 (64%)	96 (60%)
1	191 (30%)	127 (26%)	25 (36%)	64 (40%)

**Table 2:** The result of the model and the modality of input of the model in survival analysis.

Model Configuration				Testing Result (C-Index)			
Backbone	Modality	Attention	MAE	MFS	RFS	PFS	OS
**ViT-b**	PET + CT	Cross-Attention	Yes	**0.796** **(0.737, 0.851)**	**0.626** **(0.574, 0.670)**	**0.641** **(0.582, 0.690)**	**0.691** **(0.645, 0.744)**
**ViT-b**	PET	Self Attention	Yes	0.730 (0.669, 0.812)	0.576 (0.499, 0.643)	0.588 (0.506, 0.657)	0.620 (0.561, 0.682)
**ViT-b**	CT	Self Attention	Yes	0.742 (0.674, 0.819)	0.601 (0.553, 0.655)	0.623 (0.575, 0.680)	0.626 (0.571, 0.679)
**ViT-b**	PET + CT	Cross-Attention	No	0.777 (0.715, 0.839)	0.621 (0.571, 0.663)	0.630 (0.574, 0.679)	0.678 (0.637, 0.720)
**UNet**	PET + CT	Cross-Attention(Conv)	Yes	0.791 (0.735, 0.846)	0.623 (0.572, 0.669)	0.636 (0.577, 0.683)	0.682 (0.636, 0.732)

**Table 3:** Different methods of feature fusion for the application of the attention schemes.

Model and Structure Configuration			External Testing Result (C-Index)			
Q-Former	Cross Attention	Concat + FC	MFS	RFS	PFS	OS
yes	qk@CT v@PET	No	0.796 (0.737, 0.851)	0.626 (0.574, 0.670)	0.641 (0.582, 0.690)	0.691 (0.645, 0.744)
no	qk@CT v@PET	No	0.558 (0.491, 0.629)	0.525 (0.475, 0.572)	0.522 (0.474, 0.569)	0.560 (0.500, 0.611)
no Image embedding + Features	qk@CT v@PET	No	0.760 (0.702, 0.810)	0.603 (0.559, 0.652)	0.618 (0.571, 0.671)	0.667 (0.601, 0.711)
yes	qk@PET v@CT	No	0.790 (0.732, 0.844)	0.625 (0.577, 0.669)	0.638 (0.580, 0.690)	0.680 (0.635, 0.721)
yes	no	Yes	0.782 (0.728, 0.835)	0.616 (0.559, 0.661)	0.625 (0.570, 0.676)	0.678 (0.634, 0.719)

**Table 4:** The utility of different losses for experimental results.

Loss Configuration			External Testing Result (C-Index)			
Cox Loss	Proposed Loss	Focal Loss	MFS	RFS	PFS	OS
no	yes	yes	0.796 (0.737, 0.851)	0.626 (0.574, 0.670)	0.641 (0.582, 0.690)	0.691 (0.645, 0.744)
yes	no	yes	0.791 (0.731, 0.848)	0.628 (0.573, 0.674)	0.639 (0.581, 0.688)	0.687 (0.642, 0.743)
no	yes	no	0.781 (0.723, 0.835)	0.619 (0.568, 0.662)	0.630 (0.574, 0.683)	0.680 (0.637, 0.731)

**Figure A1:**
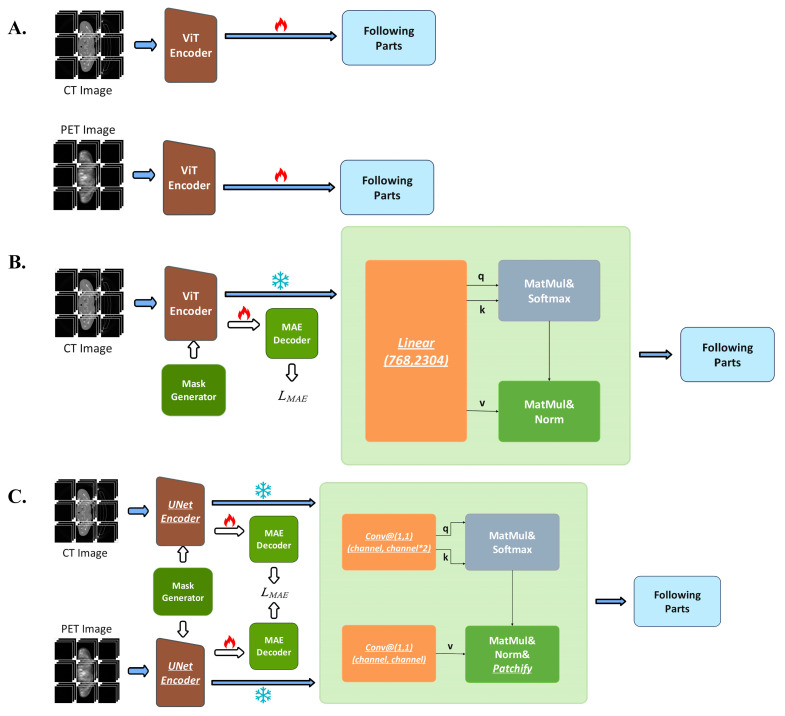
The details of the backbone models in Table 2. (**A**): the feature extractor training process; (**B**): the details of the pretrain and the following parts; (**C**): the UNet as the backbone in our pipeline.

## Data Availability

The data presented in this study are available on request from the corresponding author.

## Appendix A

1. $\lambda_{0}(t)$ was set according to the following equation and t is the time (month) in survival analysis,

$$\lambda_{0}(t)=\frac{1}{1+e^{-(t-36)/72}}$$

2. Figure A1C shows the UNetEncoder as the backbone, described in Table 2, and we replaced ViT with UNet, as shown in the above figure, ultimately confirming the superiority of using ViT as a feature extractor in the detailed comparative experiment. Figure A1A,B show the feature extractor and the muti-modal module training process in our pipeline.

3. Table A1 presents the model performance in relation to four distinct evaluation metrics (MFS, RFS, PFS, OS) from four independent medical research organizations (HGJ, HMR, TCGA, MAASTRO), each representing different medical centers. The reliability of our model’s performance has been validated and supported through the utilization of multi-center data. This inclusion of multi-center data sources contributes to the elucidation of the model’s robustness when confronted with data from diverse medical practices and patient populations.

**Table A1:** The details of the dataset and the multi-center results.

	MFS	RFS	PFS	OS
HGJ (16)	0.817 (0.760, 0.865)	0.649 (0.591, 0.692)	0.650 (0.590, 0.697)	0.699 (0.652, 0.755)
HMR (41)	0.783 (0.719, 0.844)	0.615 (0.560, 0.667)	0.634 (0.573, 0.689)	0.681 (0.634, 0.732)
TCGA (29)	0.779 (0.712, 0.843)	0.620 (0.573, 0.668)	0.622 (0.559, 0.682)	0.682 (0.631, 0.734)
MAASTRO (74)	0.813 (0.758, 0.860)	0.640 (0.579, 0.673)	0.650 (0.588, 0.696)	0.696 (0.649, 0.747)
